# Home-field advantage: native gecko exhibits improved exertion capacity and locomotor ability in structurally complex environments relative to its invasive counterpart

**DOI:** 10.1186/s12983-020-00368-8

**Published:** 2020-08-17

**Authors:** Austin M. Garner, Alexandra M. Pamfilie, E. J. Hamad, Rachael Kindig, Joshua T. Taylor, Colleen K. Unsworth, Peter H. Niewiarowski

**Affiliations:** 1 Department of Biology, The University of Akron, Akron, OH 44325-3908 USA; 2 Gecko Adhesion Research Group, The University of Akron, Akron, OH 44325-3908 USA; 3 Integrated Bioscience Program, The University of Akron, Akron, OH 44325-3908 USA; 4 Department of Education, The University of Akron, Akron, OH 44325 USA

**Keywords:** *Gehyra oceanica*, Gekkonidae, Habitat structure, *Hemidactylus frenatus*, Locomotor performance, Mo’orea, Physiology, Structural complexity

## Abstract

**Background:**

Invasive species are of substantial concern because they may threaten ecosystem stability and biodiversity worldwide. Not surprisingly, studies examining the drivers of biological invasion have increased in number over the past few decades in an effort to curtail invasive species success by way of informing management decisions. The common house gecko, *Hemidactylus frenatus*, has successfully invaded the Pacific islands where it appears to thrive in and dominate non-natural habitats offering high food availability (i.e., well-lit human dwellings) compared to native geckos. Previous work demonstrated that *H. frenatus* can outperform the native gecko, *Lepidodactylus lugubris*, in terms of maximal sprint speed on relatively simple planar surfaces (e.g., building walls). *Lepidodactylus lugubris* and other native geckos, however, may have superior locomotor performance in three-dimensional, structurally complex habitats.

**Results:**

Here we compared the locomotor behaviour and exertion capacity of the native gecko, *Gehyra oceanica*, and the invasive gecko, *Hemidactylus frenatus*, on the island of Mo’orea, French Polynesia, on fabricated structures simulating structurally complex substrates. We found that the native gecko exhibits improved locomotor performance compared to the invasive gecko on structurally complex substrates. We also completed encounter surveys to document free-ranging habitat use and behaviour of these two species. We discovered that *H. frenatus* were more common in natural habitats than previously observed and used similar substrates as *G. oceanica*, although *G. oceanica* appeared to use substrates with greater perch heights (i.e., trees).

**Conclusions:**

Our findings revealed that locomotor performance in complex environments may contribute to the previously observed habitat segregation between native and invasive Pacific island geckos. Furthermore, our locomotor and habitat use data are consistent with the hypothesis that *G. oceanica* may be resistant to invasion of *H. frenatus* in natural environments. Our study calls for more detailed ecophysiological and ecomorphological studies of both native and invasive Pacific gecko species.

## Background

Invasive species may threaten the stability of ecosystems and biodiversity worldwide [1–6]. Introduced species may be detrimental to communities or ecosystems by overutilizing local resources [7, 8], outcompeting native species [7, 9, 10], predating on organisms without adapted defences [5, 11, 12], introducing new parasites and diseases [13–16], and contributing to habitat degradation [17, 18]. These consequences cause invasive species to destabilize ecosystem interactions and functions [3, 5, 19], and, in the worst cases, drive decline or extinction of native species [2, 19, 20]. Islands are particularly vulnerable because they are more at risk for extinctions in general [21]. The common house gecko, *Hemidactylus frenatus*, is a particularly successful island invasive [4, 22, 23], spreading throughout the tropics and across multiple island systems in the Pacific [7, 22–26]. Indeed, native gecko species such as *Lepidodactylus lugubris* have been displaced from urban buildings and settlements by this invasive gecko and forced to retreat into forests and vegetated areas [22]. Several mechanisms have been proposed to explain the apparent success of *H. frenatus* worldwide, including differences in aggression, foraging strategy, physiology, and habitat structure.

Early work on the invasion success of *H. frenatus* determined that sexual *H. frenatus* individuals are more aggressive than asexual *L. lugubris* individuals, allowing them to monopolize food resources [7, 24]. As these geckos are primarily insectivorous and nocturnal, the insects that gather near artificial lights become an important food source [7, 22]. Studies suggest that *H. frenatus* outcompete and displace native geckos from urban environments where artificial lighting is plentiful [7, 27]. Previous work has also implicated variation in locomotor performance in *H. frenatus*’s relative success over their native counterparts. Work by Niewiarowski et al. [26], for example, examined differences in sprint speed and adhesive performance between *H. frenatus* and *L. lugubris*, finding no difference in adhesion but a significant difference in maximum sprint speed on artificial surfaces, such as the walls of human dwellings. *Hemidactylus frenatus* was capable of significantly higher maximum sprint speeds than *L. lugubris*, consistent with *H. frenatus*’s exploitation of food and habitat resources [7, 26]. Petren and Case [27], however, found that increasing habitat structural complexity by means of visual and spatial aluminium baffles significantly increased the success of *L. lugubris* relative to *H. frenatus*. The lack of a clear line of sight in the more cluttered, complex habitat was thought to decrease *H. frenatus*’s foraging success, perhaps negating its speed advantage [26, 27]. Structural complexity, however, also complicates effective locomotion in such environments by means of physical barriers or obstacles that must be traversed, potentially contributing to *H. frenatus*’s decreased success in structurally complex environments [26].

Here we studied the locomotor performance (locomotor behaviour and exertion capacity) of *H. frenatus* and another native gecko, *Gehyra oceanica*, on structures designed to resemble structurally complex vegetation. The inclusion or exclusion of branch points also allowed us to vary structural complexity of our models. We expected that *G. oceanica*, a gecko found primarily in forested and natural habitats [28, 29], would exhibit fewer behaviours consistent with difficulty moving in structurally complex habitats relative to *H. frenatus* on both of our structures, yet the magnitude of this difference would be greater on the branched structure than the unbranched structure. Furthermore, considering that *H. frenatus* has relatively high sprint speeds [26], we expected *H. frenatus* to have lower maximal exertion than the native gecko, *G. oceanica*, given that maximal sprint speed often trades-off with endurance capacity [30, 31]. Finally, we hypothesized that increases in structural complexity in our models would exacerbate the observed interspecific differences in exertion capacity.

Studies in various parts of its introduced range suggest that whether *H. frenatus* is capable of establishing itself beyond human dwellings and associated disturbed habitat may depend on multiple factors that vary biogeographically (see [32]). Anecdotal evidence from previous visits to Mo’orea (PHN, personal observations) suggested that *H. frenatus* was relatively uncommon, compared to *G. oceanica* in vegetated habitats not associated with buildings. We compared apparent relative abundance of *H. frenatus* and *G. oceanica* using transects to estimate encounter frequencies and select characteristics of perch types in habitats unassociated with human dwellings.

## Results

**Fig. 1:**
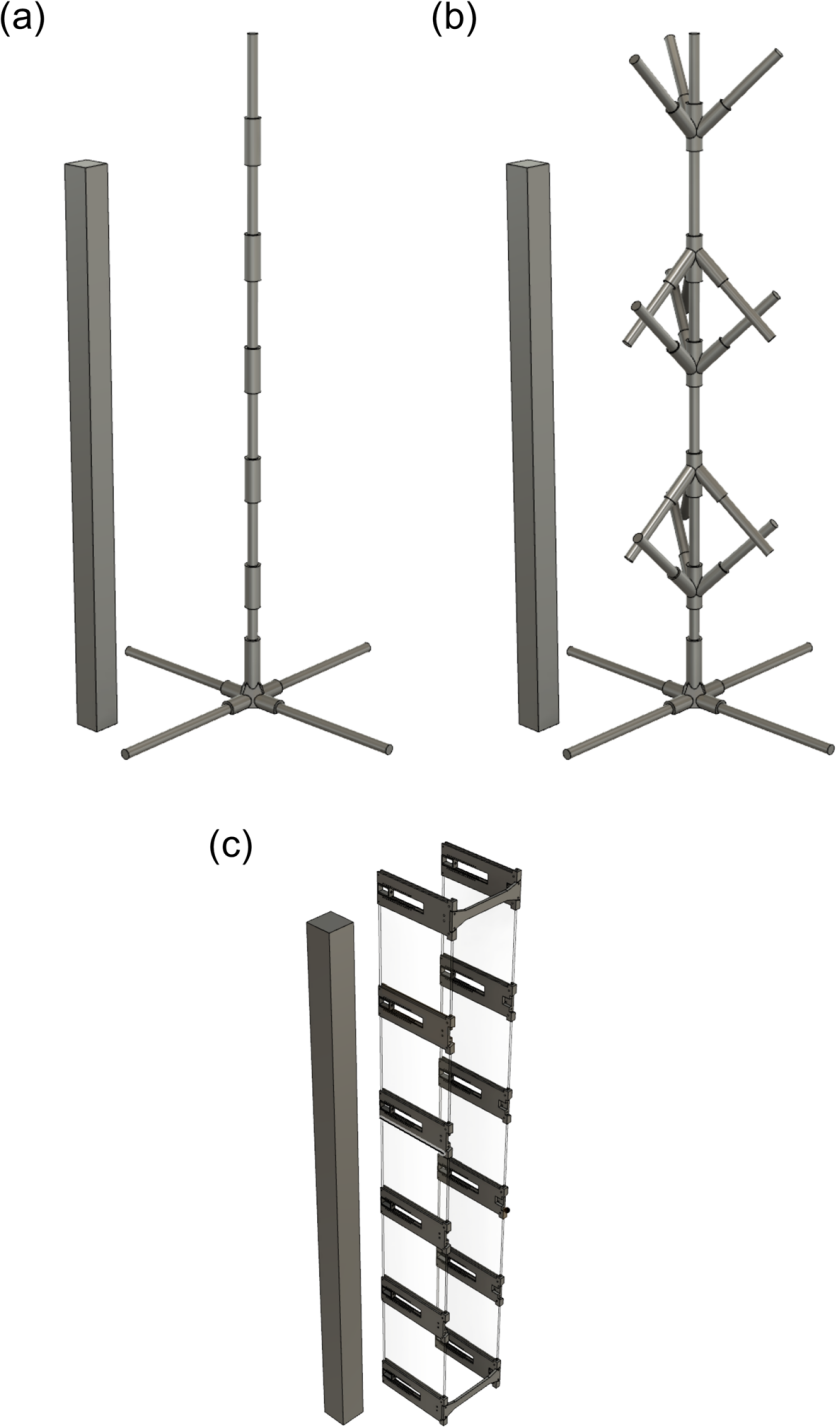
Schematics of apparatuses used in this study, including our unbranched (**a**) and branched (**b**) wooden dowel structures. We used a custom-built racetrack (**c**) equipped with IR break beams and an Arduino to measure exertion capacity and locomotor performance on a flat, two-dimensional surface (i.e., a painted wall). For scale, the structure to the left of each schematic is 1 m in height

**Fig. 2:**
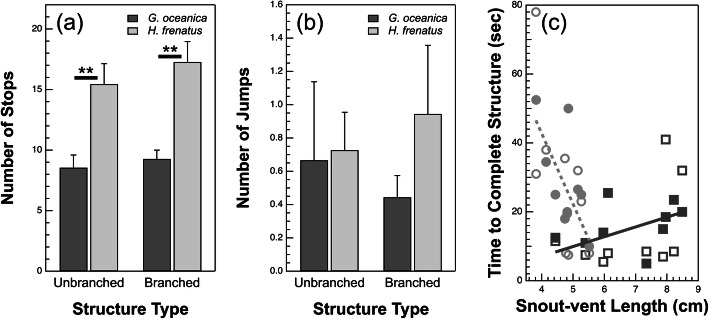
**a** Mean number of stops per run as a function of structure type and species. There were no significant differences in the number of stops per run between the unbranched and branched structures (*P* = 0.34) or the structure type-species interaction (*P* = 0.73), but *H. frenatus* stopped significantly more than *G. oceanica* on both structures (*P* = 0.0003). **b** Mean number of jumps as a function of structure type and species. The number of jumps per run did not significantly vary as a function of structure type (*P* = 0.61) or species (*P* = 0.13). **c** Mean time to complete structure (TCS) as function of snout-vent length (SVL), species (*Hemidactylus frenatus* = circles, *Gehyra oceanica* = squares), and structure type (unbranched = open symbols, branched = closed symbols). TCS did not significantly vary as a function of structure type (*P* = 0.16), species (*P* = 0.94), or their interaction (*P* = 0.88), but varied significantly with SVL (*P* = 0.037) and the interaction between species and SVL (*P* = 0.0038). Although TCS does not differ considerably between the two species relative to the variance, TCS of *H. frenatus* is strongly negatively correlated with SVL, whereas that of *G. oceanica* exhibits a slight positive relationship with SVL. ** *P* < 0.001

We collected a number of locomotor behavioural variables while native and invasive geckos were repeatedly sprinted up vertical unbranched and branched dowel structures (Fig. 1; see Methods for details). The number of stops varied significantly between species (*P* = 0.0003) but did not vary significantly between unbranched or branched structures (*P* = 0.34; Fig. 2a, Table 1, and Table 2). *Hemidactylus frenatus* stopped significantly more per run on both unbranched and branched structures compared to *G. oceanica*. The number of jumps per run were analysed for a species and structure type effect using the Wilcoxon rank sum test. The number of jumps did not significantly vary as a function of species or structure type (species: *P* = 0.13; structure type: *P* = 0.61; Fig. 2b, Table 1, and Table 2). Although this nonparametric alternative was unable to capture variance as a result of our repeated measures (individual gecko over unbranched and branched structures), there are no conspicuous effects of structure type or species relative to the variance (Fig. 2b). Time to complete structure (TCS) did not vary significantly as a function of species (*P* = 0.94) or structure type (*P* = 0.16), but varied significantly with SVL (*P* = 0.037) and the interaction between species and SVL (*P* = 0.0038; Fig. 2c, Table 1, and Table 2). Although TCS does not differ considerably between the two species relative to the variance, TCS of *H. frenatus* is strongly negatively correlated with SVL, whereas that of *G. oceanica* exhibits a slight positive relationship with SVL. We observed no significant difference in the number of branches traversed or path length travelled by the different species on the branched structure (branches: *P* = 0.10; path length: *P* = 0.086; Table 3 and Table 4). There was no significant interaction between species and substrate type in any of our analyses that included it as a factor (*P* > 0.05). The inclusion of individual gecko as a random factor explained 36 and 8% of the variance in the number of stops and TCS, respectively.

**Table 1:** Number of jumps per run, number of stops per run, number of runs, maximal exertion time (MET), and time to complete structure (TCS) as a function of structure type and species. Values are means ± s.e.m. Different letters signify significant differences between species for that particular structure

Structure type	Species	Jumps	Stops	Runs	MET (sec)	TCS (sec)
Unbranched	*G. oceanica*	0.67 ± 0.47	8.55 ± 1.04^A^	3.78 ± 0.52^A^	122.45 ± 19.10^A^	9.34 ± 2.27
	*H. frenatus*	0.73 ± 0.23	15.45 ± 1.68^B^	1.67 ± 0.26^B^	70.38 ± 21.09^B^	9.82 ± 3.57
Branched	*G. oceanica*	0.44 ± 0.13	9.28 ± 0.72^A^	3.22 ± 0.62^A^	143.01 ± 22.31^A^	12.39 ± 3.01
	*H. frenatus*	0.94 ± 0.41	17.28 ± 1.69^B^	1.17 ± 0.27^B^	72.16 ± 21.62^B^	12.36 ± 4.40

**Table 2:** Analysis of variance (ANOVA) tables for the number of jumps per run, time to complete structure (TCS), number of stops per run, maximal exertion time (MET), and the number of runs

**Number of jumps per run**				
**Source**	**S**	**Z**	**P**	
Species	302.5	−1.49	0.13	
Structure type	368.0	0.51	0.61	
*Time to complete structure (TCS)*				
**Source**	**DFNum**	**DFDen**	**F**	**P**
Species	1	14.1	0.005	0.94
Structure type	1	15.8	2.15	0.16
Species*Structure type	1	15.8	0.02	0.88
SVL	1	15.3	5.19	0.037*
Species*SVL	1	15.3	11.6	0.0038*
*Number of stops per run*				
**Source**	**DFNum**	**DFDen**	**F**	**P**
Species	1	17.4	19.97	0.0003*
Structure type	1	16.6	0.98	0.34
Species*Structure type	1	16.6	0.12	0.73
*Maximal exertion time (MET)*				
**Source**	**DFNum**	**DFDen**	**F**	**P**
Species	1	17	4.81	0.042*
Structure type	1	19	0.81	0.38
Species*Structure type	1	19	0.42	0.52
Mass	1	17	4.63	0.046*
Species*Mass	1	17	3.29	0.087
*Number of runs*				
**Source**	**DFNum**	**DFDen**	**F**	**P**
Species	1	19	24.35	< 0.0001*
Structure type	1	19	1.78	0.17
Species*Structure type	1	19	0.005	0.97

*indicates a significant effect

**Table 3:** Gecko path length and number of branches geckos traversed on the branched structure, maximal exertion time (MET) on a planar surface, the total number of stops on a planar surface, and maximum sprint speed on a planar surface. Values are means ± s.e.m.

Species	Path Length (m)	Branches	MET (sec)	Total stops	Speed (cm/sec)
*G. oceanica*	1.41 ± 0.03	2.22 ± 0.24	91.70 ± 9.00	47.5 ± 5.3	43.41 ± 20.32
*H. frenatus*	1.30 ± 0.04	1.67 ± 0.22	108.93 ± 28.8	34.87 ± 5.2	15.51 ± 34.42

**Table 4:** Analysis of variance (ANOVA) tables for the number of branches traversed and gecko path length on the branched structure, maximal exertion time (MET) on a planar surface, total number of stops on a planar surface, and maximum sprint speed on a planar surface

Source	DF	F	P
*Number of branches*			
Species	1	2.94	0.10
*Path length*			
Species	1	3.35	0.086
*Maximal exertion time (MET) on planar surface*			
Species	1	0.33	0.58
Mass	1	1.83	0.20
Species*Mass	1	0.97	0.34
*Total number of stops on planar surface*			
Species	1	2.78	0.11
*Maximum sprint speed on planar surface*			
Species	1	0.49	0.50
SVL	1	0.37	0.55
Species*SVL	1	0.99	0.34

**Fig. 3:**
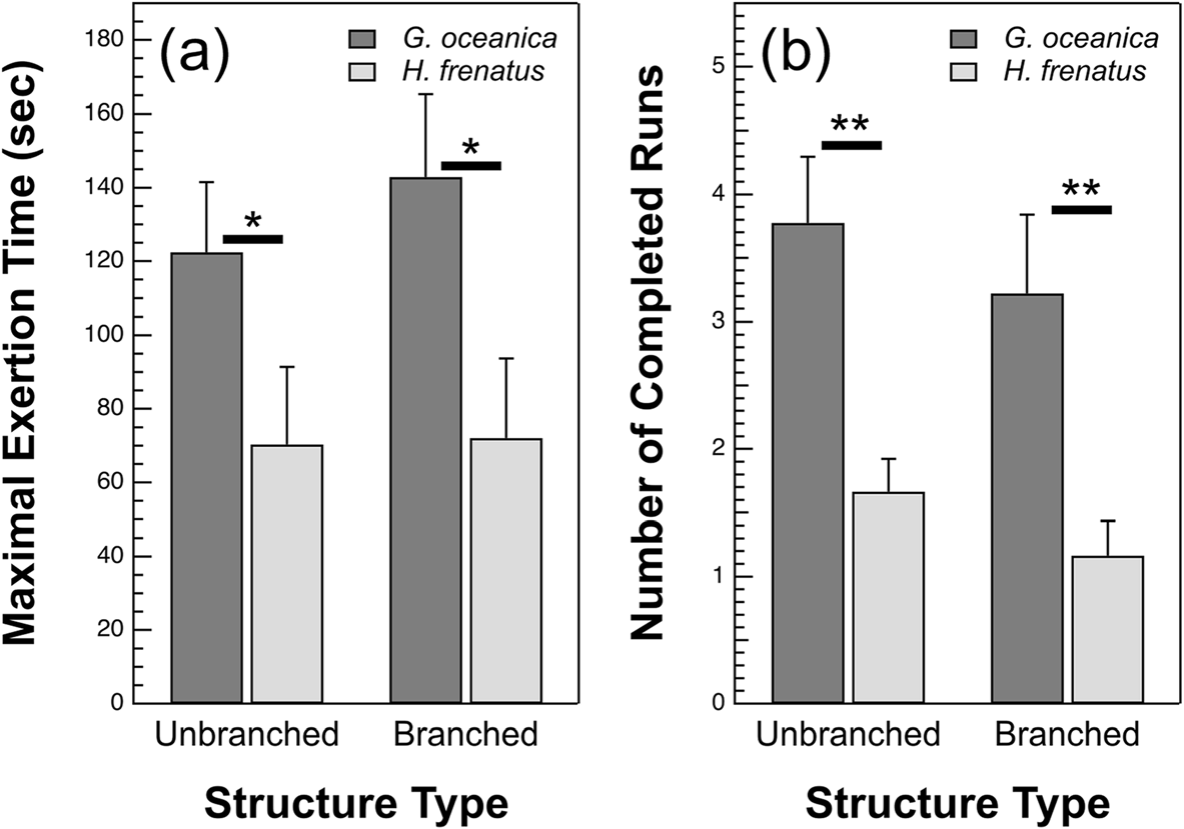
**a** Least squares mean maximal exertion time (MET) as a function of species and structure type. *G. oceanica* had a significantly longer MET than *H. frenatus* (*P* = 0.042), but there were no significant differences between structure types (*P* = 0.38) or the structure type-species interaction (*P* = 0.52). **b** Mean number of completed runs as a function of structure type and species. *G. oceanica* completed significantly more runs than *H. frenatus* (*P* < 0.0001), although there were no significant differences between structure types (*P* = 0.17) or the substrate type-species interaction (*P* = 0.97). * *P* < 0.05, ** *P* < 0.0001

**Fig. 4:**
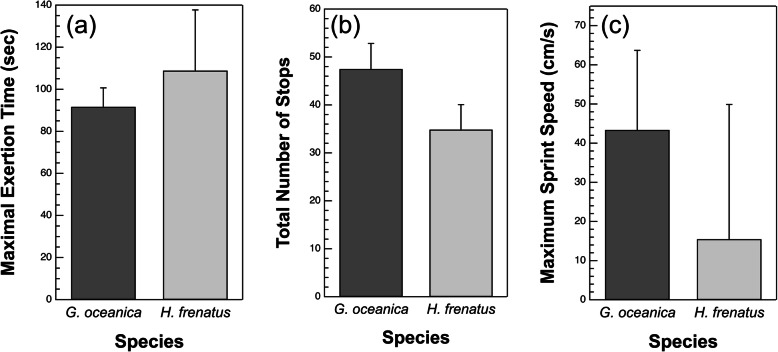
**a** Least squares mean MET on a planar, vertical surface as a function of species. There was no significant effect of species on mean MET on this surface (*P* = 0.58). **b** Total number of stops traversed on a planar, vertical surface as a function of the two species. There were no significant differences in the total number of stops between the two species (*P* = 0.11). **c** Least squares mean maximum sprint speed of the two gecko species on a planar surface. Maximum sprint speed did not significantly vary between *G. oceanica* and *H. frenatus* (*P* = 0.50)

We measured maximal exertion time (MET) for both species of gecko as they were repeatedly sprinting up our dowel structures. There was a significant difference in MET between species (*P* = 0.042; Fig. 3a, Table 1, and Table 2), but no difference between the branched and unbranched structures (*P* = 0.38). *H. frenatus* reached exhaustion significantly more quickly than *G. oceanica*. Not surprisingly, *G. oceanica* completed significantly more runs than *H. frenatus* on both structures (*P* < 0.0001; Fig. 3b, Table 1, and Table 2). Structure type had no significant effect on the number of completed runs (*P* = 0.17). We also measured MET on a planar, vertical surface and there was no significant difference in maximal exertion time between the species (*P* = 0.58; Fig. 4a, Table 3, and Table 4), suggesting there are only species effects on our unbranched and branched dowel structures. We also observed no significant difference in the total number of stops or maximum sprint speed as a function of species on the vertical, planar surface (stops: *P* = 0.11; speed: *P* = 0.50; Fig. 4b and c, Table 3, and Table 4). There was no significant interaction between species and substrate type in any of our analyses that included it as a factor (*P* > 0.05). The inclusion of individual gecko as a random factor explained 17 and 3% of the variance in MET and the number of completed runs, respectively.

Over four evenings, we located a total of 126 *G. oceanica* and 76 *H. frenatus* perching at an average of 2.1 ± 0.18 m versus 1.2 ± 0.26 m, respectively (Welch’s ANOVA: F = 37.3, *P* < 0.0001). There were no significant differences between the two species in perch or animal orientation. In general, both species were usually observed to be in the head pointing up or down direction (~ 70%) on vertical perches, which accounted for more than half of all observed perches (~ 60%). The two species did differ with respect to the proportion of observations occurring on natural (tree, leaf) versus synthetic (fence post, fence wire) substrates with *H. frenatus* more frequent on synthetic (59%) and *G. oceanica* more frequent on natural (56%) substrate perches (χ^2^ = 4.7, *P* < 0.029). The difference in frequency of observations on natural and synthetic substrates appeared to be driven by *G. oceanica* occurring on trees about twice as frequently as *H. frenatus,* leading to a significant difference in the overall distribution of observations across the four substrate types (fence post, fence wire, tree, leaf; χ^2^ = 10.8, *P* < 0.018; Table 5).

**Table 5:** % of observations in which *G. oceanica* and *H. frenatus* were observed on four different substrate types (leaf, fence wire, tree, fence post)

Substrate Type	***G. oceanica***	***H. frenatus***
Leaf	16%	20%
Fence wire	19%	18%
Tree	40%	21%
Fence post	25%	41%

Several of our analyses revealed nonsignificant effects, potentially a result of low statistical power. The use of retrospective power analysis in the interpretation of results is highly controversial (e.g., [33–35]). Steidl et al. [34], for example, note that retrospective power analyses offer no additional insight than that provided by hypothesis testing because statistical power scales with the *p*-value; nonsignificant effects will always have low statistical power. Nevertheless, retrospective estimations of statistical power given the minimum biologically relevant effect sizes are considered informative [34] and we employed this approach to examine our results. We found no significant species effects in our experiment measuring maximal exertion time (MET), the total number of stops, and maximum sprint speed on a planar surface. If species effects existed, we would have expected to find similar effect sizes to those found on our dowel structure (MET and stops) or previous work (maximum sprint speed, [26]). Species effect sizes on our dowel structures for MET and the total number of stops were reasonably large (MET Cohen’s d = 1.78; Stops Cohen’s d = 1.72), and previous work measuring maximum sprint speed between *H. frenatus* and *Lepidodactylus lugubris* on planar surfaces [26] found massive species effect sizes (Cohen’d = 3.16). If similar effect sizes were observed in our experiment on a planar surface, statistical power would have been 93% or greater, an adequate amount of statistical power to determine relevant differences between groups. Assuming that a Cohen’s d greater than or equal to 1 (i.e., the difference between means is greater than 1 standard deviation) is a biologically relevant effect size for the remainder of the behavioural and performance data, statistical power would have been at least 51% for the analysis of the number of branches/path length traversed or 85% for all other analyses (all else being equal). Therefore, we interpret the nonsignificant results in the analysis of the number of branches/path length traversed as inconclusive, calling for additional study. For the balance of the effects, we would have had adequate statistical power to detect biologically relevant differences between groups.

## Discussion

Invasive species are known to have considerable impacts on native biota, including ecological displacement as well as competition for local resources [7, 8, 23, 36]. The worldwide invasion of the common house gecko (*Hemidactylus frenatus*) is a prime example of this, as many local species have been displaced, and even brought to extinction, by this successful invasive species [22, 23, 36]. Although unfortunate, the colonization of invasive species presents a unique opportunity to investigate the mechanisms and drivers of ecological invasion [37, 38]. In this work, we examined the locomotor performance (behaviour and exertion capacity) of a native gecko (*Gehyra oceanica*) and *H. frenatus* in Mo’orea, French Polynesia on structurally complex substrates. Our work elucidates the impacts of habitat complexity on gecko locomotion and its potential relevance to the ecological displacement occurring between native and invasive Pacific island geckos.

We observed significant differences in the number of stops per run between the native and invasive gecko on both of our dowel structures. Specifically, *H. frenatus* stopped more often on both structures than *G. oceanica*. Stopping or pausing behaviours are correlated with locomotion in structurally complex habitats. *Sceloporus woodi*, for example, stop more often on vertical substrates than flat ones [39] and *S. malachiticus* pause more when tall obstacles are placed in their locomotor paths [40]. The increased number of stopping behaviours of *H. frenatus* suggests that they have greater difficulty navigating structures more closely resembling natural vegetation than planar surfaces (e.g., our dowel structures) relative to *G. oceanica*. We also measured maximal exertion time (MET) to compare exertion capacity of the species on the complex structures. *Hemidactylus frenatus* reached exhaustion significantly more quickly than *G. oceanica*. That *G. oceanica* completed a significantly higher number of successful runs on the structures suggests that the native gecko, *G. oceanica*, has a significantly higher exertion capacity than the invasive gecko, *H. frenatus*, on structures meant to mimic more natural locomotor contexts. Interestingly, we observed no significant species effects on MET, the total number of stops, and maximum sprint speed on a planar surface, suggesting that interspecific differences in the measured variables are only present on our dowel structures. Therefore, these findings are consistent with the interpretation that the complexity of the dowel structures is driving the interspecific differences in locomotor behaviour and performance.

The time to complete the structure (TCS) was significantly impacted by the interaction between species and SVL, suggesting that TCS of the two species scales differently with body size. Specifically, small increases in SVL appear to have a marked negative effect on TCS of the smaller-bodied *H. frenatus*, while larger changes in SVL have a slight positive effect on TCS of the larger-bodied *G. oceanica*. This result is unexpected because time-based performance measures should scale negatively with body size; larger organisms should complete a structure of given length more quickly than smaller ones (e.g., [41]). This appears to be the case for *H. frenatus*, but it is not clear why this is not the case for *G. oceanica*. Individual variation in performance measures can often disguise or obscure trade-offs or trends that have been observed elsewhere [42]. For example, Calsbeek and Careau [42] found that increased variability in individual performance caused significant positive relationships when negative ones would be expected. Therefore, interindividual variation in performance could have certainly led to the observed scaling of TCS with body size in *G. oceanica*.

Contrary to our initial expectations that the additional complexity via the introduction of branch points would exacerbate species differences, we observed no significant structure type effects or species-structure type interactions in any of our analyses. This suggests that the addition of branch points did not impact the locomotor behaviour or performance of either species and that both species responded similarly to the change in complexity. Of course, it is impossible to know whether the addition of branch points in our structures realistically mimics the structural complexity geckos experience when moving through natural vegetation. Thus, potential differences in locomotor behaviour and performance may be apparent in more natural circumstances. It was also surprising that we observed significant species effects in the number of stops per run but not in other measures implemented to gauge difficulty navigating our dowel structures (e.g., jumps and TCS). TCS did not differ between species because *H. frenatus* must have compensated for its increased number of stops by increasing its sprint speed. Jumps were relatively uncommon in our experiments (mean < 1 for both species on both structure types), thus it is possible that jumping is not a regular locomotor mode for these geckos during maximal exertion.

Previous studies comparing *H. frenatus* and another native gecko, *Lepidodactylus lugubris*, demonstrated that *L. lugubris* flourish in terms of body condition and foraging success in structurally complex environments. This indicated that *H. frenatus*’s advantage of greater sprint speed and foraging success in simple environments was diminished under such conditions [26, 27]. While we studied the impact of structural complexity on locomotion of *H. frenatus* and a different native gecko, our results suggest that interspecific differences in locomotor behaviour and exertion capacity in structurally complex environments may also contribute to the differential success of native and invasive species in such habitats. Specifically, we found that the invasive gecko has difficulty navigating and exhibits reduced exertion capacity on structurally complex substrates. Decreased locomotor performance could make *H. frenatus* more prone to predation events, interspecific conflict, and reduced foraging success in such environments [27]. Our results suggest the hypothesis that *G. oceanica* is resistant to substantive impacts resulting from the invasion of *H. frenatus* on Mo’orea when both are present in structurally complex environments. Indeed, one of us travelled to the same location on Mo’orea 8 years prior to this experiment and rarely observed *G. oceanica* and *H. frenatus* occupying similar habitats (PHN, personal observations). However, our encounter surveys suggest that *H. frenatus* has become quite common in habitats well removed from human dwellings. Moreover, *H. frenatus* and *G. oceanica* use perches that were very similar, differing only in the average perch height of natural substrates recorded. This may suggest that *H. frenatus* is either beginning to ecologically displace *G. oceanica* or perhaps that the two species will live in sympatry. Nevertheless, it seems incontrovertible that *H. frenatus* is likely to move into habitats suitable for other geckos like *G. oceanica* and *L. lugubris* in French Polynesia.

Of course, our study examines only one potential factor related to ecological invasion success in structurally complex natural environments (locomotor performance) and there are several other non-mutually exclusive factors that can affect this [43–45]. Urban and natural areas can differ drastically in their distribution of prey, competitors, and predators [22, 43]; urban areas, for example, generally attract greater amounts of insect prey (the major diet of these geckos) than more natural areas [7, 22, 27]. Urban and natural environments also vary in ambient conditions; urban habitats tend to be warmer and more arid than forested ones [45]. Phenotypes can also shift in response to the variability in habitat structure in urban versus natural habitats; *Anolis* from urban habitats possess greater relative limb length than forest dwelling *Anolis* and this results in greater sprint speed on artificial substrates [44, 45]. Therefore, future work investigating the invasion success of *H. frenatus* should focus on holistically quantifying and examining the relationships between morphology, performance, and ecology of both invasive and native gecko species.

## Conclusions

We examined the locomotor performance of a native and invasive gecko on the island of Mo’orea on structurally complex locomotor substrates. We found that the native gecko, *G. oceanica*, exhibits improved locomotor performance compared to the invasive gecko, *H. frenatus*, on structurally complex substrates. This suggests that *G. oceanica* may be relatively resistant to the invasion of *H. frenatus* on the island, at least within the context of locomotor performance. Future work should consider performing long-term ecological surveys of the gecko species on Mo’orea in an effort to fully document the status of their respective populations, ecological niches, and the progression of the invasive species, *H. frenatus*. Focused research on the interactions between native and invasive species, such as our study here, not only enhance our understanding of the drivers of biological invasions, but also inform management strategies that can curtail the worldwide success of invasive species.

## Methods

### Animals

The Oceania gecko (*Gehyra oceanica)* is native to the island of Mo’orea [46], while the common house gecko (*Hemidactylus frenatus*) is invasive across the Pacific islands [47]. *Hemidactylus frenatus* has been used as a species of interest in the past [26, 27] in conjunction with *Lepidodactylus lugubris*. We initially planned to complete this experiment with *L. lugubris*, however, upon arrival to our study site on Mo’orea, we had difficulty locating a sufficient number of *L. lugubris*, leading to our focus on *G. oceanica*. Past studies on other islands have indicated that these two species may occupy similar ecological niches and occur in similar proportions across habitats [29, 48, 49].

On Mo’orea, we captured 39 geckos over 3 days (19 *G. oceanica*, 20 *H. frenatus*) via noose. Geckos were marked on the dorsum at the base of the tail with a metallic permanent marker to prevent recapture. The geckos were held individually in cloth bags when not actively being used in experiments and were released within 24 h at the site of capture. Body mass and snout-vent length (SVL) were measured for each gecko using a digital balance and ImageJ, respectively (National Institutes of Health, Bethesda, MD USA, [50]). Geckos that had recently lost their tail or had poor body condition were not caught or used in trials.

### Experimental procedures

Experiments were performed on two complex, artificial structures built from 1.9 cm wooden dowel rods connected by 3D-printed polylactic acid (PLA) connectors. We characterized these structures as complex, relative to planar surfaces (e.g., building walls), because they introduced three-dimensionality and provided geckos with navigational choices as they completed locomotion trials. We also varied structural complexity by including or excluding branch points. The unbranched structure (Fig. 1a) was a single “trunk” of dowels, while the branched structure (Fig. 1b) possessed a single “trunk” with “branches”. The final height of each structure was approximately 1.25 m. The branches of the branched structure were ~ 15 cm in length and occurred at ~ 20 cm intervals.

For trials on our complex structures, nine *G. oceanica* (*n* = 9) and twelve *H. frenatus* (*n* = 12) were used. Geckos were placed at the base of the structure and prodded by the handler by gently tapping the base of the tail. If the gecko fell or jumped to the ground, it was caught and placed back at the location it exited. Once a gecko reached the top, it was placed immediately at the base of the structure to continue the trial. Geckos were chased up the structures until they would no longer move with continual prodding and were no longer able to right themselves. The time elapsed from the start of the first run up the structure until this point was recorded as maximal exertion time (MET). Trials were completed in the dark with only red headlamps for illumination because both species of gecko are nocturnal.

For the first and second runs up the structure, the total time taken to reach the top (time to complete structure or TCS) was recorded, along with the number of stops, jumps, and branches traversed (on the branched structure). A stop was defined as any time when all four feet of the gecko were in contact with the substrate and the gecko was not moving [51, 52]. A jump was defined as a deliberate leap from one place to another. The number of completed runs was also recorded. Trials were video recorded with a DSLR camera (Nikon D5600; Nikon Inc., Melville, NY USA) to determine the path of movement and estimate the total path length. Geckos were tested on both the branched and unbranched structures, and the order of the treatment (branched or unbranched) and individual gecko were randomized.

We tested for interspecific differences in maximal sprint speed and MET on a planar locomotor substrate (building wall) using an additional 18 geckos (8 *H. frenatus*, 10 *G. oceanica*). Geckos were repeatedly sprinted up a vertical, 1 m custom-built racetrack (Fig. 1c) equipped with IR break beams (Adafruit Industries, New York, NY USA) and an Arduino UNO (Arduino, Somerville, MA USA) to obtain five split time measures of sprint speed. MET was estimated as with the complex structures and total number of stops was recorded for each animal. Each animal was tested once, and the order of individual gecko was randomized. All experimental protocols were reviewed and approved by the Ministère De La Culture Et De L’Environnement, French Polynesia.

### Gecko encounter surveys

We walked a 2.4 km transect between the hours of 2000 and 2200 h local time on four consecutive evenings to survey for active geckos using a headlamp. After starting the transect, observers walked until sighting a gecko, at which time the observer stopped and completed a visual scan of the entire area for all observable geckos. The duration of scans was 100 s. The approximate perch height, substrate type (leaf, tree, fence post, fence wire), substrate orientation (horizontal, vertical, inclined), and animal orientation (head pointing up, head pointing down, sideways, horizontal) was recorded for each located animal. At the end of the scan, the observer resumed walking the transect until the next gecko was sighted, repeating the location scan and recording as previously described. The transect was a closed loop comprised of dirt road, walking trail, and paved rural road sections of approximately equal length. Except for the first and last approximately hundred meters of the transect, there were no human dwellings, except a maintenance garage, closer than 0.5 km. Approximately 500 m of the length of the transect passed through closed canopy forest, while the remainder consisted of a fence row separating pasture/plantation with sparsely distributed trees along the transect itself.

### Statistical analyses

The mean number of stops, jumps, and branches (on the branched structure) per gecko were obtained by averaging across the first two runs on the particular structure. If a particular individual was unable to complete the structure before exhaustion, only MET data was used. The fixed effects of species, structure type, and their interaction on our dependent variables were examined using a series of analyses of variance (ANOVAs) and analyses of covariance (ANCOVAs). Mixed model ANOVAs were used for the mean number of stops and number of completed runs. TCS was analysed with a mixed model ANCOVA with SVL as a covariate. Differences in gecko path length and number of branches taken on the branched structure were compared between species with univariate ANOVAs.

MET on our simulated models was compared between species, structures, and their interaction using a mixed model ANCOVA with mass as a covariate. MET on the planar, two-dimensional substrate was compared between species using ANCOVA with mass as a covariate. Total number of stops on the planar surface was analysed for a species effect using ANOVA. Maximum sprint speed on the flat substrate was calculated using the five split time measures of sprint speed. The effect of species on maximum sprint speed was examined using ANCOVA with SVL as a covariate. MET and TCS data were natural log transformed to meet the assumptions of ANOVA. These data were back transformed post hoc for data presentation purposes. The residuals of the number of jumps did not meet assumptions of normality and no transformation was able to alleviate this problem. As such, we used the nonparametric Wilcoxon rank sum test to investigate whether the mean number of jumps per run varied as a function of structure type or species. Individual gecko was modelled as a random effect in all of our mixed model analyses.

Qualitative substrate use and behavioural data obtained from gecko encounter surveys were analysed using a series of chi-square tests. Perch height was analysed using Welch’s ANOVA because variance between groups was not homogeneous. All statistical tests were conducted using JMP Pro 14 (SAS Institute Inc., Cary, NC USA).

## Data Availability

The dataset supporting the conclusions of this article is available in the figshare repository, 10.6084/m9.figshare.12616175.

## References

[1] Sakai AK, Allendorf FW, Holt JS, Lodge DM, Molofsky J, With KA (2001). The population biology of invasive species. Annu Rev Ecol Syst.

[2] Clavero M, García-Berthou E (2005). Invasive species are a leading cause of animal extinctions. Trends Ecol Evol.

[3] Reaser JK, Meyerson LA, Cronk Q, Poorter MD, Eldrege LG, Green E (2007). Ecological and socioeconomic impacts of invasive alien species in island ecosystems. Environ Conserv.

[4] Fisher RN (2011). Considering native and exotic terrestrial reptiles in island invasive species eradication programmes in the tropical Pacific.

[5] Doherty TS, Dickman CR, Nimmo DG, Ritchie EG (2015). Multiple threats, or multiplying the threats? Interactions between invasive predators and other ecological disturbances. Biol Conserv.

[6] Richardson DM, Pyšek P (2008). Fifty years of invasion ecology – the legacy of Charles Elton. Divers Distrib.

[7] Petren K, Case TJ (1996). An experimental demonstration of exploitation competition in an ongoing invasion. Ecology..

[8] Funk JL, Vitousek PM (2007). Resource-use efficiency and plant invasion in low-resource systems. Nature..

[9] Tsutsui ND, Suarez AV, Holway DA, Case TJ (2000). Reduced genetic variation and the success of an invasive species. P Natl A Sci USA..

[10] Sax DF, Stachowicz JJ, Brown JH, Bruno JF, Dawson MN, Gaines SD (2007). Ecological and evolutionary insights from species invasions. Trends Ecol Evol.

[11] Blackburn TM, Cassey P, Duncan RP, Evans KL, Gaston KJ (2004). Avian extinction and mammalian introductions on Oceanic Islands. Science..

[12] Doherty TS, Glen AS, Nimmo DG, Ritchie EG, Dickman CR (2016). Invasive predators and global biodiversity loss. P Natl A Sci USA.

[13] Wikelski M, Foufopoulos J, Vargas H, Snell H. Galápagos birds and diseases: invasive pathogens as threats for island species. Ecol Soc. 2004;9(1):1–0.

[14] Fisher MC, Garner TWJ (2007). The relationship between the emergence of *Batrachochytrium dendrobatidis*, the international trade in amphibians and introduced amphibian species. Fungal Biol Rev.

[15] Clark NJ, Olsson-Pons S, Ishtiaq F, Clegg SM (2015). Specialist enemies, generalist weapons and the potential spread of exotic pathogens: malaria parasites in a highly invasive bird. Int J Parasitol.

[16] Miaud C, Dejean T, Savard K, Millery-Vigues A, Valentini A (2016). Curt grand Gaudin N, et al. invasive north American bullfrogs transmit lethal fungus *Batrachochytrium dendrobatidis* infections to native amphibian host species. Biol Invasions.

[17] Gurevitch J, Padilla DK (2004). Are invasive species a major cause of extinctions?. Trends Ecol Evol.

[18] Didham RK, Tylianakis JM, Hutchison MA, Ewers RM, Gemmell NJ (2005). Are invasive species the drivers of ecological change?. Trends Ecol Evol.

[19] Murray J, Murray E, Johnson MS, Clarke B (1988). The extinction of *Partula* on Moorea. Pac Sci.

[20] Lowe S, Browne M, Boudjelas S, De Poorter M. 100 of the world’s worst invasive alien species: a selection from the global invasive species database. Vol. 12. Invasive Species Specialist Group Auckland; 2000.

[21] MacArthur RH, Wilson EO (1963). An equilibrium theory of insular zoogeography. Evolution..

[22] Case TJ, Bolger DT, Petren K (1994). Invasions and competitive displacement among house geckos in the tropical Pacific. Ecology..

[23] Cole NC, Jones CG, Harris S (2005). The need for enemy-free space: the impact of an invasive gecko on island endemics. Biol Conserv.

[24] Bolger DT, Case TJ (1992). Intra- and interspecific interference behaviour among sexual and asexual geckos. Anim Behav.

[25] Rödder D, Solé M, Böhme W. Predicting the potential distributions of two alien invasive Housegeckos (Gekkonidae: *Hemidactylus frenatus*, *Hemidactylus mabouia*). North-West J Zool. 2008;4(2):236–46.

[26] Niewiarowski PH, Stark A, McClung B, Chambers B, Sullivan T (2012). Faster but not stickier: invasive house geckos can out-Sprint resident mournful geckos in Moorea. French Polynesia J Herpetol.

[27] Petren K, Case TJ (1998). Habitat structure determines competition intensity and invasion success in gecko lizards. P Natl A Sci USA..

[28] Reeder NMM (2005). The effects of competition on the distributions of three gecko species on Moorea, French Polynesia.

[29] Wiles GJ, Rodda GH, Fritts TH, Taisacan EM (1990). Abundance and habitat use of reptiles on Rota. Mariana Islands Micronesica.

[30] Huey RB, Bennett AF, John-Alder H, Nagy KA (1984). Locomotor capacity and foraging behaviour of Kalahari lacertid lizards. Anim Behav.

[31] Vanhooydonck B, Damme RV, Aerts P (2001). Speed and stamina trade-off in Lacertid lizards. Evolution..

[32] Hoskin CJ (2011). The invasion and potential impact of the Asian house gecko (Hemidactylus frenatus) in Australia: Asian House Geckos Invading Australia. Austral Ecol.

[33] Thomas L (1997). Retrospective power analysis. Conserv Biol.

[34] Steidl RJ, Hayes JP, Schauber E. Statistical power analysis in wildlife research. J Wildlife Manage. 1997;61(2):270–9.

[35] Gerard PD, Smith DR, Weerakkody G. Limits of retrospective power analysis. J Wildlife Manage. 1998:801–7.

[36] Dame EA, Petren K (2006). Behavioural mechanisms of invasion and displacement in Pacific island geckos (*Hemidactylus*). Anim Behav.

[37] McNeely J (2001). Invasive species: a costly catastrophe for native biodiversity. Land Use and Water Resources Research.

[38] Losos JB. Lizards in an evolutionary tree: ecology and adaptive radiation of anoles. Berkeley: Univ of California Press; 2011.

[39] Higham TE, Korchari P, McBrayer LD (2011). How to climb a tree: lizards accelerate faster, but pause more, when escaping on vertical surfaces. Biol J Linn Soc.

[40] Kohlsdorf T, Biewener AA (2006). Negotiating obstacles: running kinematics of the lizard *Sceloporus malachiticus*. J Zool.

[41] Huey RB, Hertz PE (1984). Effects of body size and slope on acceleration of a lizard (*Stellio* (agama) *stellio*). J Exp Biol.

[42] Calsbeek R, Careau V (2019). Survival of the fastest: the multivariate optimization of performance phenotypes. Med Sci Sports Exerc.

[43] Stroud JT, Colom M, Ferrer P, Palermo N, Vargas V, Cavallini M (2019). Behavioral shifts with urbanization may facilitate biological invasion of a widespread lizard. Urban Ecosyst.

[44] Winchell KM, Maayan I, Fredette JR, Revell LJ (2018). Linking locomotor performance to morphological shifts in urban lizards. P Roy Soc B-Biol Sci.

[45] Winchell KM, Reynolds RG, Prado-Irwin SR, Puente-Rolón AR, Revell LJ (2016). Phenotypic shifts in urban areas in the tropical lizard *Anolis cristatellus*. Evolution..

[46] Fisher RN (1997). Dispersal and evolution of the Pacific Basin Gekkonid lizards *Gehyra oceanica* and *Gehyra mutilata*. Evolution..

[47] Petren K, Bolger DT, Case TJ (1993). Mechanisms in the competitive success of an invading sexual gecko over an asexual native. Science..

[48] Pearlman LH (2014). Resource partitioning and food preference of three Gekkonid species on the island of Moorea, French Polynesia.

[49] Sabath MD (1981). Gekkonid lizards of Guam, Mariana Islands: reproduction and habitat preference. J Herpetol.

[50] Schneider CA, Rasband WS, Eliceiri KW (2012). NIH image to ImageJ: 25 years of image analysis. Nat Methods.

[51] Garner AM, Stark AY, Thomas SA, Niewiarowski PH (2017). Geckos go the distance: Water’s effect on the speed of adhesive locomotion in geckos. J Herpetol.

[52] Stark AY, Ohlemacher J, Knight A, Niewiarowski PH (2015). Run don’t walk: locomotor performance of geckos on wet substrates. J Exp Biol.

